# The evolution of Indian psychiatric research: An examination of the early decades of the Indian Journal of Psychiatry

**DOI:** 10.4103/0019-5545.69199

**Published:** 2010-01

**Authors:** Rajiv Radhakrishnan, Chittaranjan Andrade

**Affiliations:** Department of Psychiatry, Schizophrenia Research Clinic, Yale University School of Medicine, New Haven, Connecticut, USA-06511; 1 National Institute of Mental Health and Neurosciences, Bangalore - 560 029, India

**Keywords:** Indian Psychiatric research, Research methodology, Indian Journal of Psychiatry

## Abstract

Research in psychiatry has travelled far since the inception of the Indian Journal of Psychiatry (IJP) in 1949. We reviewed publications in the IJP during its initial three decades to identify path breaking articles and trends in research. We present the evolution of research design in the IJP from cases studies to randomized controlled trials. We identify the earliest studies in different fields, ranging from drug trials to social interventions, and from women’s mental health to geriatric psychiatry. We consider special issues such as the measurement of psychopathology specific to the Indian context, studies of treatments specific to Indian traditions, epidemiology of psychiatric disorders in India, and innovations in service delivery. Students interested in the history of Indian psychiatric research will be rewarded by the richness and variety of thought evidenced in the publications in the early decades of the IJP.

“… in truth, knowledge doesn’t suddenly appear in neat and tidy quanta.

Like patches of lichen spreading across a rock face, it accretes over decades.”

*Goeff Watts*[1]

## INTRODUCTION

Milestones in science are achievements in research that are recognized once they have been attained and after, often long after, they have been passed. Indian research in the past was no different from what it is today: Marked by a multitude of ideas, numerous lines of research, and varied methodological approaches; and milestones were what happened, as it were, almost by chance.

A milestone is a landmark; were there landmarks in the publications in the Indian Journal of Psychiatry (IJP)? Perhaps not; at least not in the sense of landmarks that stand out in the history of psychiatry. However, there were indeed sustained fields of enquiry and important transitions in research, both of which marked the growth of the journal across five decades of publication. This article seeks to illustrate the process of maturation of the journal from its inception in 1949 to the present year, 2009, through an examination of publications in selected domains of investigation. The emphasis is on research published during the initial two to three decades; that is, the pre-DSM-III era. Brief reference will also be made to more recent publications.

## METHODS IN RESEARCH

Most of the papers published in the journal during the 1950s were theoretical in nature. These were characteristically biased towards psychoanalysis and ‘depth psychology’, such as in the description of common obsessions and compulsions,[2] Indian concepts concerning the development of the psyche,[3] the role of aggression in emotional development[4] and a psychoanalytical view of human personality.[5]

The first formal piece of research published in the journal was an observational study of the “Psyche of the Tuberculous” in 400 ex-soldiers at the Military Hospital in Aundh.[6] The first systematic interventional study was a case series of histamine and insulin treatment of schizophrenia.[7] The study included 20 patients with schizophrenia, 17 of whom were followed up for 4-9 years. The author noted that the response in early cases of simple and catatonic schizophrenia was encouraging. The first trial of an investigational drug was a small study of four patients with ‘mental deficiency’, conducted at the Inter-Provincial Mental Hospital, Kanke. In this study,[8] 1.7- 3.4 kg of glutamic acid was administered at a dose of 15-30 g/d across 6-12 months. Other than an increase in weight, which couldn’t be attributed only to glutamic acid, no change was observed in motor activity, intelligence, or clinical functioning.

Examples of other early therapeutic trials were an examination of the use of sodium pentothal in narcotherapy,[9] a study of intensified electroconvulsive therapy (ECT) where the initial shock at 150 V was followed by seven additional shocks during the fit[10] and an investigation of insulin sub-coma treatment in schizophrenia and anxiety states.[11] There were no control groups in these early studies; and in some studies, sample sizes were very small, often comprising just 4-10 patients.[8,12,13]

The first randomized, double-blind, placebo-controlled trial in the world was published on October 30, 1948.[14] The first study of a similar nature to appear in the IJP addressed the efficacy of Guaiacol Glycerol Ether (GGE) in anxiety states.[15] For this study, placebo tablets were made of the same size, color, shape, and taste as GGE; and as GGE is bitter, quinine was added to placebo tablets. The authors initially assigned patients by alternation, not randomization, to drug vs. placebo; subsequently, using a crossover design, they shuttled patients between drug and placebo, week by week, from the start of the study to the 6-week endpoint. Possible carryover benefits were not considered; improvement in anxiety without separation of GGE from placebo was attributed to the establishment of rapport; in other words, a psychodynamic explanation was offered for a placebo effect!

Menon *et al*.,[16] presented one of the first efforts at matching experimental and control groups in a study of prochlorperazine vs. placebo in women with chronic schizophrenia; however, the two groups were managed in separate wards and not in the same environment. Prochlorperazine was observed to outperform placebo.

Notably, in some regards Indian researchers kept abreast with the Western world. For example, results of transorbital leukotomy[17,18] appeared within five years of the introduction of this technique by Freeman.[19] A therapeutic trial of primidone with patients serving as their own controls was published within two years of the introduction of the drug.[20]

The 1970s witnessed a sharp increase in the report of randomized, double-blind drug trials with active or placebo comparator arms. Important trials examined flupenthixol,[21] fluphenazine enanthate,[22] iprindole,[23] lorazepam,[24] prothipendyl,[25] thiothixene,[26] trifluoperidol,[27] trimipramine,[28] dibenzepin[29] and trioxizine.[30] Today, of course, randomized controlled trials (RCT) are the de facto standard of research in the journal.

### Describing psychopathology

In an early study of psychiatric phenomenology, Mukherji[31] presented a psychoanalytic explanation of hallucinations in two patients. Amazingly, academic deterioration as a prepsychotic manifestation of schizophrenia was studied in a large series as early as during the 1950s;[32] however, the study lacked operational definitions and a control group. Another important paper[2] dealt with the cultural determinants of the content of obsessional symptoms in Indian patients; however, the paper was impressionistic and lacked empirical support. Hoch[33,34] also examined cultural content in a phenomenological study of depression. These early investigations paved the way towards the study of Indian elements in psychiatry.

During the 1970s, there was a spurt in phenomenological studies with efforts to explain the form and content of symptomatology in relation to Indian culture. These studies addressed disorders such as schizophrenia,[35] acute and transient psychosis,[36] depersonalization,[37] hysterical psychosis,[38] possession,[39] keemam dependence,[40] cannabis psychosis,[41] dhat syndrome[42] and others. Other studies addressed the effect of Indian religions on mental health and illness,[43] attempted an Indian classification of psychiatry disorders,[36] utilized Indian mythological concepts in psychotherapy.[43,44] Attempts were also made to develop Indian systems for assessment and treatment, as will be discussed in later sections.

### Measuring psychopathology

The first effort at deriving Indian norms for an instrument was that of Asthana,[45] for the Rorschach. The first effort to devise a new scale was described by Murthy;[46] this study addressed communication disturbances in schizophrenia. This study is significant because the first full working draft of the Present State Examination with a scale to grade speech appeared in 1967.[47] The first study to use a validated rating scale to measure psychopathology appeared in 1966;[48] the tool was the Hamilton Rating Scale for Depression.

Though the introduction of psychometric tools designed by Indian investigators[49,50] appeared elsewhere, the first scale to appear in the IJP was in 1973; this was a neuroticism scale in Hindi.[51] Today there are more than three hundred Indian versions of scales and tests available to the mental health professional (http://www.mohfw.nic.in/listfac/PsychiatryR/List%20of%20Psychological%20Tests%20and%20Apparatus.doc), including vernacular translations of the Eysenck Personality Inventory,[52] PGI Memory Scale,[53] The Depression-Happiness Scale,[54] Psychoticism Scale In Hindi,[55] Presumptive Stressful Life Events Scale (PSLES),[56] Amritsar Depressive Inventory,[57] Burden Assessment Schedule,[58] Complex passage test for logical memory[59] and a Life Events Scale for Armed Forces Personnel.[60]

### Research into biological underpinnings of psychiatric treatment

The search for a biological basis for psychiatric disorders marked a turning point in psychiatric research. Shah and Desai[61] reported three patients who presented with features of catatonic schizophrenia but who turned out to have pontine hemorrhage with cerebral infarction, left frontal lobe abscess with pontine infarct, and left frontal lobe abscess, respectively. Rao *et al*.,[62] reported a study of absolute eosinophil cell counts (then considered an indirect measure of adrenocortical function) in patients with depression. They found no predictable relationship between eosinophil count and severity of depression, or between agitated and retarded forms of depression.

The first study to use the electroencephalography (EEG) as an investigational tool appeared in the Indian Journal of Neurology and Psychiatry in 1952: Davis and Mookerjee[63] reported that there was no significant relationship between clinical improvement with ECT (Electro Convulsive Therapy) and the degree to which delta activity replaced beta activity in the EEG.

Other studies explored the role of histamine and histaminases in schizophrenia,[64] the role of plasma cortisol in depression,[65] the association of schizophrenia with ABO blood group,[66] the relationship between urinary 5-hydroxyindoles and depression[67] and the role of HLA Class-I antigens in delusional disorder,[68] to name a few of the biological studies.

Recent studies have tried to understand psychiatric disorders using newer tools, such as spectrophotometric assay in relating serum dopamine beta hydroxylase with neurological soft signs and tardive dyskinesia,[69] Brainstem Auditory Evoked Response in opioid dependence,[70] computer tomography (CT) study to assess ventricular brain ratio and correlate it with neurological soft signs and cognitive dysfunction,[71] P300 Event-related potential in healthy controls and in patients with depression,[72,73] magnetic resonance imaging (MRI),[74] EEG fractal dimensions and spectral power analysis in prediction of antidepressant response to unilateral ECT,[75] Positron Emission Tomography study in schizophrenia,[76] and others.

### Research into Indian forms of treatment

The isolation of Reserpine from the herb Rauwolfia Serpentina[77] marks a milestone for not just Indian psychiatry but for psychiatry the world over. The observation of depression as a side-effect of reserpine paved the way for the formulation of the monoamine hypothesis for depression.[78,79]

Ray[80] and De[81] reported the use of Rauwolfia sepertina as a tablet form in two manic patients and two patients with paranoia. In 1952, Ray PK[12] described six cases treated with Rauwolfia serpentina and reported that it had beneficial effects on mental symptoms and that the total root powder is more effective than the alcoholic extract. These early studies are significant in the light of the fact that the first RCT (Randomized Controlled trial) of Rauwolfia serpentina was conducted in 1957.[82]

An interesting study explored the efficacy of an ethanolic extract of Papspalam scrobiculatum, (a variety of millet grain known as ‘Harik’ in Marathi and ‘Mino Kodro’ in Gujarati) in the management of aggressive behavior.[83] The extract was found to effect a modest improvement in psychomotor activity with a relapse of symptoms on discontinuation of the drug.

Ayurvedic preparations have also been studied in a double-blind design. Mahal *et al*., 1976, described the use of a single drug, Tagara and a combination Brahmyadiyoga compared to Chlorpromazine and placebo in the treatment of schizophrenia;[84] Centella asiatica (Mandookaparni) was evaluated in mental retardation with encouraging results.[85] Andrade *et al*.,[86] evaluated the efficacy of a herbal formulation, Memorin in age-related cognitive decline and found that Memorin resulted in significant improvement in performances on many neuropsychological tests, though improvement in many of the memory tasks was confined to males. The anxiolytic properties of an ethanolic extract of Withania somnifera (Aswagandha) was studied in a double-blind randomized control trial.[87] The authors concluded that the ethanolic extract of Withania somnifera was well-tolerated and had useful anxiolytic potential. More recently, Andrade *et al*.,[88] have demonstrated the anti-amnestic properties of Brahmi and Mandookaparni in an animal model.

Yoga has attracted worldwide interest during recent years. Vahia *et al*.,[89] devised a therapy based on Indian concepts of mental health consisting of five steps of Asana, Pranayama, Pratyahara, Dharana and Dhyana. The therapy was found to show encouraging results in a pilot study.[89] The results were better when all the five steps were involved rather than just the physical aspects of Asana and Pranayama[90] and comparable to drug therapy.[91] Yoga was also found to improve psychophysiological parameters as measured by heart rate, blood pressure, galvanic skin resistance and choice reaction time.[92]

Ayurveda and Yoga are unique contributions of Indian psychiatry to the field of Complementary and Alternative medicine.

### Studies on the distribution and determinants of psychiatric disorders

Studies on the epidemiology of psychiatric disorders were conducted by Surya *et al*.,[93] Dube,[94] Sethi, Gupta, and Kumar,[95] Sethi *et al*.,[96] and Verghese and Beig.[97]

The early pioneering studies of the distribution and determinants of psychiatric disorders are commendable for the sheer scale on which they were conducted. The first large-scale epidemiological survey in the IJP was by Dube[94] on a sample comprising about 29,000 people in and around Agra. This paper is an interim analysis of the larger study that was published in 1970.[98] In an analysis of 7,479 persons consisting of 1,333 families, lifetime prevalence of any mental disorders was reported to be 17.42/1000 (psychosis = 6.93/1000, psychoneurosis with psychopathy = 7.72/1000, mental deficiency = 2.77/1000) for rural areas and 20.19/1000 in semi-rural areas (psychosis = 5.77/1000, psychoneurosis with psychopathy = 11.54/1000, mental deficiency = 2.88/1000). Overall prevalence rates were reported as 18.32/1000 (psychosis = 6.55/1000, psychoneurosis and psycho pathy = 5 8.96/1000, mental deficiency = 2.81/1000) challenging the notion that psychosis accounted for only 2 to 3 per thousand of population. In the semi-rural population, the psychoneurosis and psychopathy seemed to show a higher trend.

The studies on suicide have been important in our current understanding of how suicide, and hence suicide prevention, in low and middle-income countries, such as India, differs from the West.[99,100] Epidemiological studies concerning the prevalence of suicide were conducted at Madurai.[101] and at Delhi.[102] The first study to appear in the IJP was in 1965.[103] The study was an analysis of 114 medical admissions into medical and pediatric wards at Erskine Hospital, Madurai over a six-month period in 1964. The figure for attempted suicide of approximately 19 per month was comparable with the average suicide frequency of 20 cases per month in Madurai. The study revealed that organophosphorus compound was the commonest agent used for suicide attempts; physical and mental illnesses and socioeconomic factors were important determinants and the lack of social cohesion with the breakup of the joint family system tended to favor suicidal attempts.

The epidemiological studies of the IPSS, DOSMeD and SOFACOS deserve mention. The International Pilot Study of Schizophrenia (IPSS),[104] the Determinants of Outcome of Severe Mental Disorders (DOSMeD) study[105] and the multi-site Study of Factors Affecting the Course and Outcome of Schizophrenia (SOFACOS)[106] served to place India on the global map with convincing evidence for a better outcome in India than in the West.

Other interesting studies in related fields in the IJP have addressed varied topics such as a study of the association between social class and mental illness,[107,108] social phenomena such as industrialization and migration,[109] Naxalite movement in Kolkata;[110] and studies on knowledge, attitude and practices, such as studies on family planning and contraception,[111] traditional healing practices[112] and community perception of delivery of mental healthcare services.[113]

### Innovations in service delivery

A shift in focus in service delivery from being treatment-centric to being patient-centric is evidenced in the pages of the Indian Journal of Psychiatry. Dube[114] describes a bold and interesting experiment made at the Agra Mental Hospital when all wards were kept open and all ward locks were removed from the charge of the attendants at a psychiatric center where traditionally, strict rules existed with regard to locking and opening hours due to an apprehension of escape, violence and other undesirable incidents. The author reported an improvement in disturbing behavior and aggression, improved sanitary conditions and decreased use of hypnotics and tranquilizers. There was also a greater turnover of patients with more admissions and discharges and more vacant beds available.

The concept of psychiatric camps is unique to the Indian setting and may be seen as the beginning of community psychiatry in India. The first psychiatric camp was held at Bagalkot on 27 August 1972 with the assistance of Friends’ Club, a Rotary Club. The paper[115] revealed that the idea was favorably accepted by general practitioners and social agencies; and was able to reach out to a substantial proportion of patients who were never-treated.

Wig *et al*.,[116] describe a model for rural psychiatric services at Raipur Rani as part of a WHO International Collaborative Study. This marks a significant shift in focus towards community psychiatry and the utility of integrative multidisciplinary approach incorporating the priorities of all stakeholders. The development of research instruments, development of tools for screening and evaluation of attitudes of the community and preparation of manuals, formation of a Mental Health Association by village leaders and utility of peripheral health workers in implementing mental health programs were significant contributions to community psychiatry.

This may be seen as a significant step forward and one that has led to the evolution of community outreach programs in its present form such as the Home Care Program.[117]

### Focus on sub-populations

In this sub-section we draw attention to the earliest publications in the IJP that dealt with specific psychiatric populations. The emergence of sub-specialties in psychiatry in India is a trend that is likely in the foreseeable future as it has done in the West and these studies mark the very beginning of this evolution.

The first paper with a special focus on woman’s health appeared in 1969.[118] The study evaluated the important causal factors of puerperal psychosis. The study found that women in the younger age groups, i.e.15-20 years, coming from rural areas, belonging to lower or lower middle class income group, and usually in their first and second para were most prone to develop puerperal psychosis. The presence of sepsis in 76% of cases was then thought to be an important factor.

The first paper on geriatric psychiatry was an assessment of psychiatric disorders in those aged 50 and above among the attendances at the Department of Psychiatry, Erskine Hospital Madurai, India.[119] The study revealed a male preponderance among the elderly psychiatrically ill. Though the pattern of prevalence of psychiatric disorders was not found to be different from the Western population, there were certain phenomenological differences, namely the paucity of depression among those with arteriosclerosis and the rarity of the ideas of guilt and sin in depressives.

The first paper on forensic psychiatry to appear in the IJP was an interesting one. D’Netto[120] studied 100 sailors in the detention quarter of the Indian Navy. One hundred offenders were divided into two groups; one with psychological treatment during punishment and the other with punishment alone. The study reported that the group that was given some psychiatric assistance along with punishment did better than the group that was given punishment alone.

The first study on child psychiatry[121] described the breakup of cases seen at a child clinic. Among the 70 children between 3-14 years, 25 were diagnosed with ‘mental deficiency’, 11 with post-encephalitic sequelae, 20 with ‘conversion hysteria’, nine with ‘behavior problem’ and one each with schizophrenia, habit disorder and epilepsy. The study identified inconsistent disciplining being more common in those with conversion ‘reactions’.

## CONCLUDING NOTES

We have sought in this article to present the diversity of processes through which Indian psychiatric research matured through the early decades of the Indian Journal of Psychiatry to its present level of advancement. Our perspective is admittedly idiosyncratic; we described what caught our attention but acknowledge that other equally meritorious fields of investigation may not have found inclusion in this paper. We hope that our descriptions will stimulate an interest in the history of psychiatric research in India, and a greater interest in the early decades of publication of the Indian Journal of Psychiatry.
